# Host and Microbiome Interplay Shapes the Vaginal Microenvironment

**DOI:** 10.3389/fimmu.2022.919728

**Published:** 2022-06-28

**Authors:** Myoung Seung Kwon, Heung Kyu Lee

**Affiliations:** Graduate School of Medical Science and Engineering, Korea Advanced Institute of Science and Technology (KAIST), Daejeon, South Korea

**Keywords:** vaginal microbiome, lactobacillus, glycogen, estrogen, bacterial vaginosis, STI, preterm birth, probiotics

## Abstract

The female reproductive tract harbors a unique microbiome, especially the vagina. The human vaginal microbiome exhibits a low diversity and is dominated by *Lactobacillus* species, compared to the microbiome of other organs. The host and vaginal microbiome mutually coexist in the vaginal microenvironment. Host cells provide *Lactobacillus* glycogen as an energy source, and *Lactobacillus* produce lactic acid, which lowers vaginal pH thereby preventing growth of other bacteria. Bacterial vaginosis can modulate host immune systems, and is frequently associated with various aspects of disease, including sexually transmitted infection, gynecologic cancer, and poor pregnancy outcomes. Because of this, numerous studies focused on the impact of the vaginal microbiome on women`s health and disease. Furthermore, numerous epidemiologic studies also have demonstrated various host factors regulate the vaginal microbiome. The female reproductive tract undergoes constant fluctuations due to hormonal cycle, pregnancy, and other extrinsic factors. Depending on these fluctuations, the vaginal microbiome composition can shift temporally and dynamically. In this review, we highlight the current knowledge of how host factors modulate vaginal microbiome composition and how the vaginal microbiome contributes to maintaining homeostasis or inducing pathogenesis. A better understanding of relationship between host and vaginal microbiome could identify novel targets for diagnosis, prognosis, or treatment of microbiome-related diseases.

## Introduction

The presence of vaginal microbiota was first observed in 1892, when German obstetrician/gynecologist Albert Döderlein identified a gram-positive, non-spore forming rod in the vaginal fluid (1). This bacterium was called *Döderlein`s bacillus* at the time of its discovery, but it was later renamed *Lactobacillus* due to its ability to produce lactic acid. Döderlein also discovered *Döderlein`s bacillus* had an antagonistic action on *Staphylococcus* growth and the absence of *Döderlein`s bacillus* in vaginal fluid was associated with puerperal fever (2, 3). These fundamental findings formed the basis of our current knowledge that *Lactobacillus* is the most prevalent bacteria in vagina and is central for women`s health. With the introduction and rapid advancement of high throughput sequencing methodologies, it is now widely accepted that the vaginal microbiome is more diverse and highly dynamic than previously assumed (4–6). Furthermore, numerous observational studies have highlighted that a non-*Lactobacillus* dominant vaginal microbiome is correlated with various diseases, including sexually transmitted infections (STIs), gynecologic cancer, infertility, and preterm birth (7–12). However, most of these correlational results were not supported by experimental study using animal models due to the uniqueness of human vaginal microbiome. This lack of experimental evidence makes it difficult to develop new diagnostic and therapeutic tools. Thus, there is an urgent need to understand mechanisms underlying the association between vaginal microbiome and its related diseases. In this review, we discuss current understanding and gaps in knowledge regarding the vaginal microbiome, focusing on host factors that change vaginal microbiome composition and how vaginal microbiome affects host reproductive homeostasis and disease.

## Vaginal Microbial Community

A thorough characterization of the optimal/healthy bacterial community is a fundamental issue in vaginal microbiome research. It was initially thought the vaginal microbiome simply consisted of *Lactobacillus*. Following the introduction of high throughput sequencing methods, it is now well established that the vaginal microbiome is more diverse than previously thought (4, 5, 13). Critically, the vaginal microbiome composition dynamically changes in response to various intrinsic and extrinsic factors (5, 6). Many scientists have tried to define optimal vaginal microbial composition despite tremendous difficulty due to the dynamic nature of the vaginal microbiome. In 2011, Ravel et al. profiled the microbial composition of healthy 396 north American women in reproductive age (4). In this cohort, five major bacterial communities were identified. Four of Community-State Types (CSTs) were dominated by *Lactobacillus* species (CST-I; *L. crispatus*, CST-II; *L. gasseri*, CST-III; *L. iners*, CST-V; *L. jensenii*), whereas CST-IV was characterized by a high diversity and high proportions of anaerobes, including *Prevotella, Dialister, Atopobium, Gardnerella, Megasphaera, Peptoniphilus, Eneahtia, Eggerthella, Aerococcus, Finegoldia*, and *Mobiluncus*. Gajer et al. further divided CST-IV into CST-IVA and CST-IVB according to abundance of *Lactobacillus* and other anaerobes (CST-IVA; moderate *Lactobacillus* abundance, CST-IVB; high abundance of *Atopobium, Prevotella, Parvimonas, Sneathia, Gardnerella, Mobiluncus, and Peptoniphilus*) (5). This CST nomenclature is still widely used. In the cohort from Ravel`s report, the vaginal microbiome of 75% of women are dominated by *Lactobacillus* species (4). In other words, the vaginal microbiome of the remaining 25% of women was classified as CST-IV, despite being healthy. This finding is remarkable because it indicates that diverse vaginal microbiome is not always associated with the disease status.

## Host Factors Determining Vaginal Microbial Community

**Figure 1:**
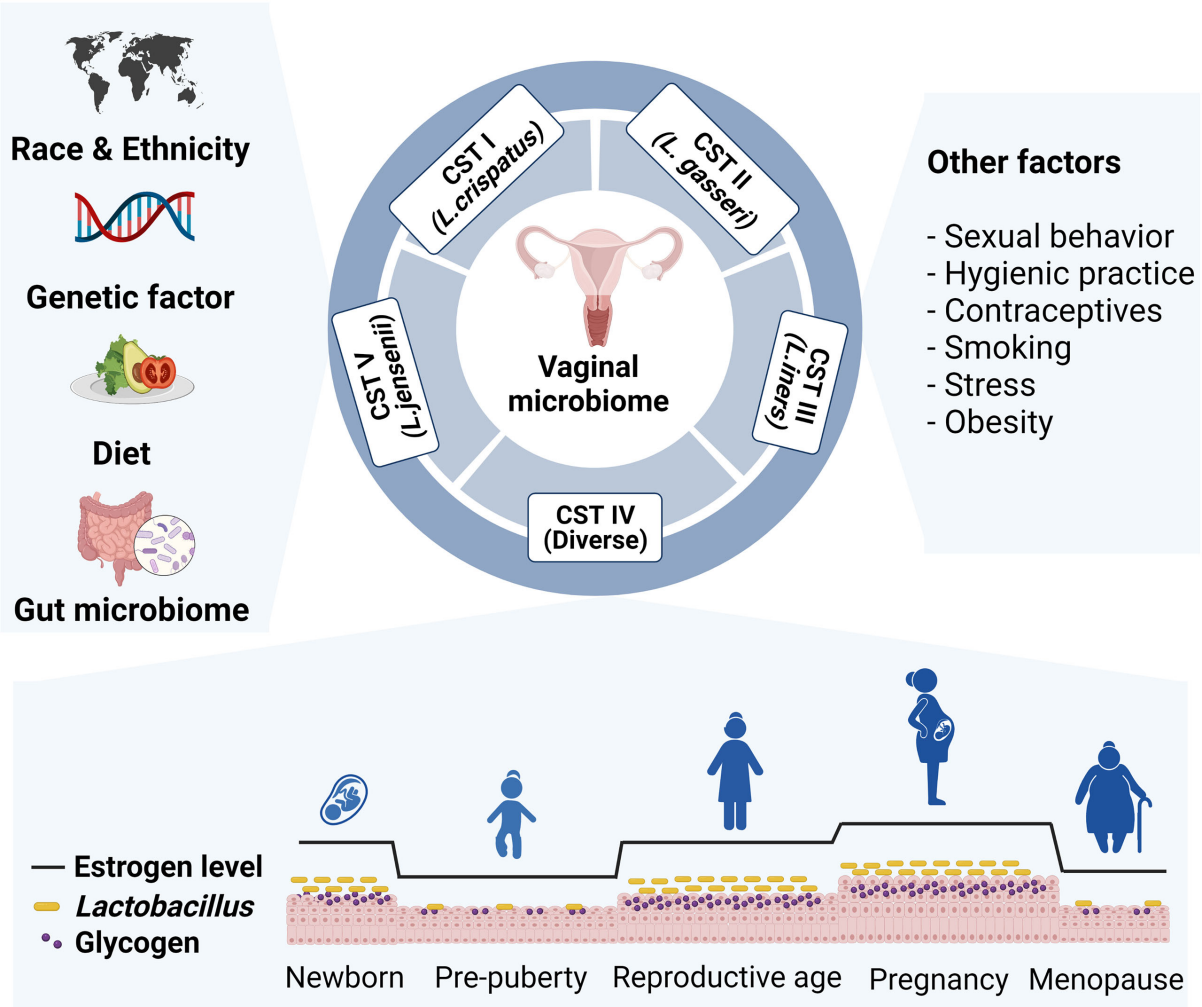
Various intrinsic and extrinsic factors that affect vaginal microbial community. The composition of the vaginal microbiome differs across host predisposing factors, such as race, ethnicity, and genetic variation. Lifestyle factors including diet, sexual behavior, hygienic practice, contraceptives, smoking, stress, and obesity also affect vaginal microbiome composition. Particularly, *Lactobacillus* abundance changes by level of glycogen, which deposits in epithelial cells upon estrogen stimulation. Prepubertal girls and postmenopausal women have relatively low abundance of lactobacillus species.

The vaginal microbiome composition varies across hosts depending on predisposing factors and physiologic status. In addition, vaginal microbiome can be altered by lifestyle factors, including diet, sexual activity, hygienic practice, antibiotics use, contraceptives, smoking, stress, and obesity (**Figure 1**) (14–18). Next, we will discuss several key factors that may impact the vaginal microbial community.

### Race, Ethnicity, and Genetic Factors

A large body of evidence has demonstrated the composition of vaginal microbiome differs across races and ethnicities. In addition to reporting on classifying CSTs in the vaginal microbiome, Ravel et al. also reported that black and Hispanic women were more likely to have CST-IV than white and Asian women (4). These results were further supported by the Human Microbiome Project (HMP) (19). In this study, European ancestry women were more likely to have a *Lactobacillus* dominant community, whereas African ancestry women tended to harbor a diverse microbial community, which was characterized by a high prevalence of *Gardnerella vaginalis* and bacterial vaginosis associated bacteria (BVAB). Differences across ethnicity were observed even at the *Lactobacillus* species level. *Lactobacillus iners* is the most common species in African ancestry women and *Lactobacillus crispatus* is the most common species in the vagina microbiome of European ancestry women (4, 13, 19). These differences across races and ethnicities suggest host genetic factors regulate the composition of the vaginal microbiome. Indeed, several studies have suggested there is a relationship between host genetic factor and microbial composition. An analysis of the vaginal microbiome in Korean monozygotic (MZ) twins, dizygotic (DZ) twins, and their families revealed the vaginal microbiomes of MZ twin pairs were more similar than DZ twins and cohabiting families. Subsequent analysis revealed Prevotella sp. was the most heritable taxa, and variations in the *IL-5* gene were associated with Prevotella sp. heritability (17). In similar study with American MZ and DZ twin women, *Lactobacillus crispatus* was heritable among European ancestry, but not African ancestry (17). Another study reported an association between genetic factors and vaginal microbiome composition in Kenyan women (20). The authors proposed that genetic variations associated with innate immune system and cell signaling could shape the vaginal microbiome composition. We can gain insight into the host genetic factors that affect the vaginal microbiome composition from these studies. However, the specific genetic variations and the mechanisms by which they may determine the vaginal microbiome composition remain unclear.

### Hormonal Changes and Pregnancy

There are several hypotheses to explain why the human vaginal microbiome is unique and dominated by *Lactobacillus*. Some of these hypotheses are mechanistic-based, whereas others focus on evolution (21). Though the existing hypotheses cannot clearly explain the unique nature of the human vaginal microbiome, it is widely accepted that estrogen and glycogen deposition in the vaginal epithelium are key factors. The vaginal mucosa of reproductive women is covered by a stratified multilayered squamous epithelium (22). Vaginal epithelial cells deposit intracellular glycogen following estrogen stimulation. Furthermore, humans have a relatively higher concentration of glycogen in vagina than other mammals (21). Although *Lactobacillus* cannot directly metabolize glycogen, α- amylase in the human genital tract can cleave glycogen into smaller carbohydrates, such as maltose, maltotriose, maltopentaose and maltodextrins, which *Lactobacillus* use as an energy source (23). During metabolic processes, *Lactobacillus* produces lactic acid and creates an acidic vaginal microenvironment (pH < 4.5) (24, 25). Consequently, other bacterial growth is suppressed (26). Because estrogen is essential for inducing *Lactobacillus* dominancy, many researchers have investigated the effects of hormonal changes on the vaginal microbiome from various perspectives.

The vaginal microbiome composition fluctuates in response to hormonal changes throughout women`s life. Because maternal estrogen passes through the placenta into the blood stream of the fetus, reproductive organs of the newborns are affected by maternal estrogen during the early days after birth (27). During this short period, the newborn vaginal epithelium reflects the glycogen content of an adult (28). After the maternal estrogen effects disappear, the concentration of vaginal glycogen becomes low until puberty. Therefore, it is likely the vaginal microbiome of prepubertal girls are not dominated by *Lactobacillus* species. In fact, an early cultivation method study demonstrated that vaginal microbiota of prepubertal girls mainly consisted of *Staphylococcus epididermis, Enterococci, Escherichia coli* (aerobic microbe), *Peptococcus*, and *Peptostreptococcus* (anaerobic microbe) (29). A recent 16s rRNA sequencing study revealed the vaginal microbiome of healthy prepubertal girls was dominated by *Prevotella, Porphyromonas*, and *Ezakiella* (30). Similar to prepubertal period, vaginal glycogen levels in menopausal women are lower than in premenopausal women (31). In line with this, the vaginal microbiome of postmenopausal women is also characterized by a low abundance of *Lactobacillus* species and high diversity (32). In a study of 87 American women, menopausal status and CST were closely associated, and postmenopausal women were more likely classified as CST-IVA (33). Critically, *Lactobacillus* loss following menopause could be restored by hormonal replacement therapy, demonstrating the importance of estrogen in regulating vaginal microbiome composition (18).

Even in reproductive aged women, the vaginal microbiome is affected by various physiologic changes, such as menstrual cycle and pregnancy. Several longitudinal studies demonstrated temporal dynamics during menstrual cycle. In a notable study, Gajer et al. analyzed vaginal swab samples from 32 reproductive aged women obtained twice weekly for 16 weeks (5). Despite individual differences, the vaginal microbiome composition rapidly changed throughout hormonal cycle in some participants. This vaginal microbiome instability was most notable during menses. Another longitudinal study using polymerase chain reaction reported similar results, that the vaginal microbiome was highly dynamic during menstrual cycle. In this study, *Gardnerella vaginalis* distinctively increased during menses. The authors hypothesized that iron enrichment following erythrocyte lysis is associated with increased *Gardnerella vaginalis* abundance (6).

Contrary to dynamic change observed throughout the menstrual cycle, the vaginal microbiome is relatively stable during pregnancy. Romero et al. longitudinally evaluated vaginal samples from non-pregnant women and pregnant women who delivered at term without complication. The authors demonstrated bacterial communities of pregnant women had a greater *Lactobacillus* abundance and were more stable compared to non-pregnant women. Although there were some microbial community transitions in pregnant women, microbial community of pregnant women often convert from a *Lactobacillus* dominated CST to another *Lactobacillus* dominated CST, not to the high diversity CST-IV (34). HMP data also revealed the vaginal microbiome was stable and dominated by *Lactobacillus* during pregnancy (35). Interestingly, microbiome stability and *Lactobacillus* abundance of pregnant African ancestry women were prominent during second and third trimesters, which are characterized by high estrogen levels. This finding also suggests a positive correlation between estrogen and *Lactobacillus* abundance.

Because the balance of estrogen and progesterone is important for maintaining female reproductive homeostasis, the effects of progesterone on the vaginal microbiome have been reported. One study of Kenyan women demonstrated depot-medroxyprogesterone acetate (DMPA) injection reduced total bacterial load and *Gardnerella vaginalis* abundance (36). Moreover, in reproductive aged women, DMPA and localized progesterone contraceptive also reduced *Lactobacillus* abundance (37, 38). Progesterone inhibits vaginal epithelium proliferation (39). Thus, lack of epithelial-derived glycogen in a progesterone-enriched environment may reduce lactobacillus and other bacterial abundance (40). Contrary to these findings, vaginal progesterone did not alter the vaginal microbiome or *Lactobacillus* abundance in pregnant women (41). Due to increased levels of endogenous estrogen and progesterone during pregnancy, the contributions of exogenous progesterone may be obscured in pregnant women. Taken together, several studies have reported progesterone reduces the vaginal microbial burden yet, how endogenous and exogenous progesterone affect vaginal microbiome remains unclear.

### Crosstalk Between the Gut Microbiome and Diet

As described above, estrogen and glycogen are important for *Lactobacillus* dominancy. One researcher hypothesized that a high starch diet increases vaginal glycogen (21). Though contrary hypotheses have been proposed. For example, another researcher hypothesized a high fat diet is associated with *Lactobacillus* dominancy because a high fat diet increased serum estradiol (42). Neither hypothesis is widely accepted due to the lack of experimental evidence. Nonetheless, dietary intake seems to be an important factor in composing microbial communities. The gut microbiome composition is strongly correlated with various nutrients from diet, such as protein, fat, sugar, starch, and fiber (43–46). Such gut microbiome alterations due to specific diets has an enormous impact on human health and disease pathogenesis (47). Although the underlying mechanism is not clearly defined, the vaginal and gut microbiomes seem to be closely related. For instance, several studies suggested the gastro-intestinal tract may serve as a bacterial reservoir or origin for the vaginal microbiome (48, 49). Furthermore, the risk of BV (Bacterial vaginosis) is correlated with *Lactobacillus* or BVAB colonization in gut (49, 50). Because of this crosstalk between the gut and vaginal microbiomes, researchers have tried to demonstrate the direct and indirect influence specific dietary nutrients may have on the vaginal microbiome.

An early study reported a correlation between dietary nutrients and BV risk based on Nugent score (51). The authors demonstrated high fat intake was associated with severe BV. Another report revealed high glycemic load was correlated with BV risk based on Nugent score (52). More recently, 16s rRNA sequencing has been used to assess the effect of specific nutrients on the vaginal microbiome (37). In this study, no single nutrient (including sugar, fiber, fat, and glucose) affected the vaginal microbiome. However, vegetarian participants had a vaginal microbiome characterized by higher alpha diversity compared to non-vegetarian participants. Taken together, these results suggest it is possible that nutrients regulate energy metabolism, which may influence vaginal microbiome (37), although it is unclear which specific nutrient is involved. Because most of these studies evaluated nutrient intake through participant`s self-assessment, cautious interpretation is necessary. Thus, a new experimental model that precisely controls specific nutrients is urgently needed to address the connection between ingested nutrients and the vaginal microbiome.

There are reports that micronutrients also affect the vaginal microbiome. Although initial reports revealed an association between many micronutrients, such as calcium, folate, β-carotene, and vitamins, with vaginal microbiome composition or BV risk (51, 53), subsequent studies did not support these results (54). Among these micronutrients, many researchers still focus on vitamin D. An early observational study reported serum level of 25-hydroxy-vitamin D [25(OH)D] was negatively corelated with BV during the first trimester (55). The association between vitamin D deficiency and BV during pregnancy was confirmed in multiple reports (56, 57). Recently, Jefferson et al. analyzed vaginal 16S rRNA profiles and serum 25(OH)D levels of pregnant women (58). The authors found a positive correlation between *Lactobacillus crispatus* and serum 25(OH)D among European ancestry. Conversely, among African ancestry, participant with lower serum 25(OH)D were more likely to have higher *Megasphaera*. However, correlation between vitamin D and the vaginal microbiome is controversial in non-pregnancy women (59, 60).

### Other Life Style Factors

Various lifestyle factors can affect vaginal microbiome composition. Cigarette smoking has been strongly associated with BV prevalence, even after adjusting other factors (19, 61). In a pilot study analyzing vaginal microbiome in smokers and non-smokers, women with CST-IV were 25-fold more likely to be smokers than women with CST-I (15). The authors further evaluated this result by performing a follow-up study investigating metabolomic profiling (62). They suggested vaginal biogenic amines, including agmatine, cadaverine, putrescine, tryptamine, and tyramine, may contribute to vaginal microbiome modulation. Further experimental evidence is needed to support this hypothesis. Several epidemiologic studies have shown that BV incidences increase as psychological stress increases (63, 64). In an early study investigating hormonal effects on glycogen deposition in the vaginal epithelium, Wrenn et al. reported the stress hormone cortisol inhibited glycogen deposition when administered with estrogen (65). Although this result was derived from rodent model with a higher cortisol concentration than is observed physiologically, this study suggests stress hormones may play an important role in vaginal glycogen deposition. Further studies are needed to evaluate the effects of stress-induced cortisol levels on human vaginal microbiomes.

Further, sexual behavior may directly impact colonization of the vaginal microbiome. Numerous epidemiologic studies have demonstrated that certain sexual behaviors were associated with BV. In a longitudinal cohort study of 773 sexually active women, BV acquisition was associated with the frequency of vaginal intercourse and the number of sexual partners (61). Moreover, condom use was protective against BV (66), suggesting that sexual activity may directly alter the vaginal microbiome composition.

Vaginal hygienic practices also impact the vaginal microbiome composition. Specifically, washing practices are associated with BV acquisition and reduced *Lactobacillus* abundance (67, 68). In one study investigating the effect of vaginal hygiene products on vaginal microbiome found *Lactobacillus* growth was not inhibited when using vinegar and iodine based products (69). Instead of directly inhibiting bacterial growth, these products induced epithelial cell death and proinflammatory response *in vitro*, marked by increased interleukin (IL)-6 and IL-1β. More investigations are needed to determine whether hygienic practices directly change the vaginal microbiome composition or indirectly change the vaginal microenvironment.

## Influence of the Vaginal Microbiome on Host Health and Disease

### Host Immune System and Modulation by the Vaginal Microbiome

**Figure 2:**
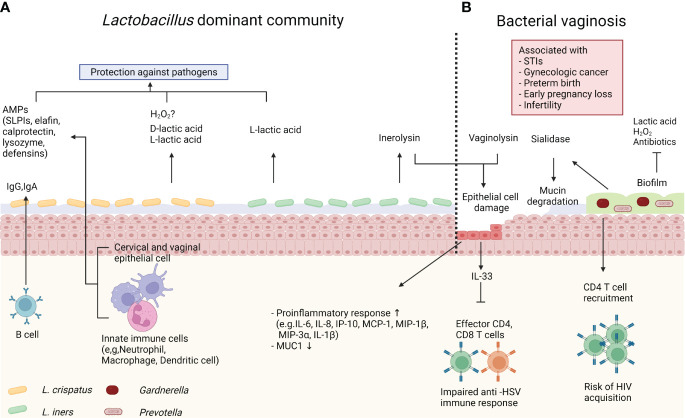
Host immune system and microbiome. **(A)** *Lactobacillus crispatus* produce both L- and D-lactic acid to protect the host against pathogens. *Lactobacillus crispatus* also can produce hydrogen peroxide (H2O2) *in vitro*, however it’s antimicrobial role *in vivo* is controversial. Innate immune cells and epithelial cells produce antimicrobial peptides (AMPs), such as secretory leukocyte protease inhibitors (SLPIs), elafin, calprotectin, lysozyme and defensins. Immunoglobulin (Ig)G and IgA from memory B cells also contribute to protection. *Lactobacillus iners* produce only L-lactic acid, and inerolysin from *Lactobacillus iners* can damage host epithelial cells. **(B)** Bacterial vaginosis (BV) is associated with various gynecologic and obstetric diseases. In BV, the vaginal microbiome is dominated by diverse anaerobe bacteria, including *Gardnerella* and *Prevotella*. These bacteria form biofilm, which confers resistance to antibiotics, lactic acid, and H_2_O_2_. BV-associated bacteria also produce sialidase and vaginolysin. Sialidase cleaves mucin, and vaginolysin damages host epithelial cells. BV induces proinflammatory response. IL-33 produced by epithelial cells directly inhibits effector CD4 and CD8 T cells during herpes simplex virus (HSV)-2 infection. Meanwhile, BV increases predisposing CD4 T cells and increases risk of human immunodeficiency virus (HIV) acquisition.

Like other mucosal tissues, the female reproductive tract (FRT) is a major portal for various pathogens. Both host and microbiota play critical roles in the protection against foreign pathogens (**Figure 2A**). The lower FRT, which consists of the ectocervix and vagina, is covered by multilayered stratified squamous epithelium on top of the lamina propria. The outermost superficial layer contains dead flattened cells, called cornified cells (70). Cornified cells are loosely attached to the epithelium and are consequently exfoliated. These exfoliated cells may act as a decoy for pathogens (22). A multilayered squamous epithelium is converted into simple columnar epithelium in the transformation zone, which under constant hormonal regulation. Above the transformation zone, the endocervical epithelium can act as a glandular tissue. Particularly, cervical epithelial cells within a crypt produce mucus, though human vaginal epithelial cells do not produce mucus unlike rodents (71). However, the human vaginal epithelium is covered with mucus produced by the cervical epithelium. The mixture of mucus and cornified cells acts as a primary physical barrier to protect the host from pathogens. Furthermore, mucus can serve as a chemical barrier. Vaginal epithelial cells and various immune cells produce antimicrobial peptides (AMPs), including secretory leukocyte protease inhibitors (SLPIs), elafin, calprotectin, lysozyme and defensins (72). It was reported that SLPIs inhibited HIV (Human immunodeficiency virus) infection of monocytes *in vitro* (73). In addition, women diagnosed with STIs, including HIV, *Neisseria gonorrhoeae*, *Trichomonas vaginalis*, *Chlamydia trachomatis*, and *Candida*, had lower SLPIs levels in their vaginal fluid (74, 75), demonstrating that SLPIs play an important role in STIs protection. Calprotectin and lysozymes, which are mainly produced by myeloid cells such as neutrophils, have direct antimicrobial activities. Calprotectin inhibits bacterial growth by iron chelating (76), and lysozymes degrade bacterial cell walls (77). Meanwhile, defensins have direct antimicrobial activity and inhibit bacterial toxins (78). Because mucus is more than 90% water, it can contain and deliver the above-mentioned water-soluble immune mediators (79). Another component of mucus is immunoglobulin (Ig). IgA is generally the most abundant antibody isotype in mucosal fluids such as saliva, tears, milk, and gastrointestinal fluid (80). However, cervico-vaginal mucus contains more IgG than IgA at different stages of the hormonal cycle (80, 81). In a mouse model, luminal IgG is produced by migrant memory B cells in the vagina rather than circulation (82). Although the role of vaginal IgG has not been fully elucidated, it was reported that IgG traps virus and protects host from viral infection (83).

*Lactobacillus*, a representative species of optimal vaginal microbiome, contribute to the hosts immunity. As mentioned previously, *Lactobacillus* produces lactic acid, and lowers vaginal pH (24, 25). The acidic microenvironment prevents growth of pathogenic bacteria, including *Chlamydia trachomatis*, *Neisseria gonorrhoeae*, and *Escherichia coli* (84–86). Additionally, lactic acid has been reported to offer protection against viral infection. Lactic acid abolished the surface charge of HIV (87). Consequently, HIV diffused slowly and got trapped in acidic mucus. Not only does lactic acid act as an acid, but it also exhibits direct antimicrobial properties. One study used a fluorescence assay to evaluate the effect of lactic acid on the outer membrane permeability of gram-negative bacteria, which revealed lactic acid had more permeabilization potency than hydrogen chloride at same pH (88). Another traditional role of *Lactobacillus* is the production of hydrogen peroxide (H_2_O_2_). Many early studies reported that the presence of H_2_O_2_-producing *Lactobacillus* species was associated with decreased risk of gynecologic and obstetric diseases (89, 90). Although H_2_O_2_ inhibited the growth of pathogenic strains, such as *Escherichia coli, Prevotella*, and *Gardnerella*, *in vitro* (91), the role of H_2_O_2_ *in vivo* is still under debate (92). For example, *Lactobacillus* needs oxygen to produce H_2_O_2_ even though *Lactobacillus* can grow in anaerobic conditions. However, human vaginal microenvironment is physiologically hypoxic (93). Critically, measured *in vivo* H_2_O_2_ levels are lower than the potential bacteriocidic level (26, 94). Moreover, H_2_O_2_ derived from *Lactobacillus* is inactivated by cervico-vaginal fluid, which is physiologic content in FRT, and semen (94). Due to these results, the antimicrobial role of H_2_O_2_ *in vivo* remains controversial.

*Lactobacillus iners* has several special properties compared to other *Lactobacillus* species. Lactic acid exists as L- and D- isomers in the vagina and it was reported D-lactic acid has more protective potency against uropathogens (95). However, *Lactobacillus iners* can produce only L-lactic acid, whereas *Lactobacillus crispatus* and *Lactobacillus gasseri* produce both D- and L- isoforms (96). Meanwhile, *Lactobacillus iners* can produce inerolysin, a member of the cholesterol-dependent cytolysin family (97).. This cytolysin can lyse eukaryotic cells, including host epithelial cells. It has been proposed that *Lactobacillus iners* lyse cells to acquire nutrients (98). Particularly, inerolysin gene expression is upregulated in dysbiosis (99). In line with this, *Lactobacillus iners* is more correlated with BV than other *Lactobacillus* species. Multiple observational studies demonstrated *Lactobacillus* iners can coexist in CST-IV with other BV-related bacteria, unlike other *Lactobacillus* species (4, 13). Furthermore, microbial communities dominated by *Lactobacillus iners* are unstable and prone to transition to CST-IV (5). In accordance with these unique features, *Lactobacillus iners* is considered as an intermediate risk bacterium.

BV is the disequilibrium status of vaginal microbiome, which is characterized by low abundance of optimal *Lactobacillus* species and increased non-optimal anaerobic species, including *Gardnerella, Prevotella, Atopobium*, and other BVAB (72). In the condition of BV, the host immune system is altered by various mechanisms (**Figure 2B**). A recent study using three-dimensional culture of human cervical epithelium demonstrated host immune responses are modulated by the microbiome (100). In this study, BVAB colonization in cervical epithelial cells induced proinflammatory response, as measured by increased expressions of IL-6, IL-8, interferon gamma induced protein (IP)-10, monocyte chemotactic protein (MCP)-1, macrophage inflammatory protein (MIP)-1β, MIP-3α, and IL-1β. MUC1 (gene encoding mucin) expression was decreased in *Gardnerella vaginalis* colonization. These findings are supported by previous epidemiological reports that revealed proinflammatory cytokines and chemokines levels are low in vaginal fluid of women with a *Lactobacillus* dominant microbiome (101, 102). In addition to immune modulation, BV can disrupt protective immune barriers. For example, mucin is a major structure for maintaining the viscosity of cervico-vaginal mucus. Mucin is a large glycoprotein composed of a long protein backbone with O-linked glycan terminated by a sugar unit, such as sialic acid (103). Several species of *Gardnerella* and *Prevotella*, which are representative bacteria of BV, produce the mucin degrading enzyme sialidase (104, 105). Bacterial sialidase cleaves terminal sialic acid, and some bacteria use the resulting carbon skeleton as an energy source (106). Thus, abnormal watery discharge in BV could be linked to mucin degradation (107). In synergic effect with mucin degradation, *Gardnerella vaginalis* may directly damage epithelial cells. *Gardnerella vaginalis* produces vaginolysin, a member of cytolysin family, and vaginolysin lyse epithelial cells in a CD59 molecule-dependent manner (108). Also, a recent study demonstrated *Gardnerella* induces human vaginal epithelial cell apoptosis (109). Another influence of BV is biofilm formation. Biofilm is a community of bacteria encapsulated in self-produced extracellular matrix that adheres to epithelium (110). Specifically, *Gardnerella vaginalis* is one of the predominant bacteria in BV-associated biofilm (111). The virulent property of biofilm is derived from its resistance to immune defense systems. In fact, *Gardnerella vaginalis* in biofilm is resistant to lactic acid and H_2_O_2_ produced by *Lactobacillus* (112). Moreover, biofilm reduces the efficacy of antibiotics, such as metronidazole (113). BV exhibits a high recurrence rate after antibiotic treatment (114). Thus, biofilm may contribute to BV recurrence.

### The Vaginal Microbiome and Sexually Transmitted Infections

The human vagina is a portal of entry for a myriad of foreign pathogens. Particularly, the FRT are exposed to pathogens during sexual activity. Both bacteria (*Chlamydia, Mycoplasma, Trichomonas*, and *Neisseria gonorrhoea* etc.) and viruses (Herpes simplex virus; HSV, HIV, Human papilloma virus; HPV etc.) can be transmitted during sexual intercourse, and these infectious diseases are referred as STIs. Numerous epidemiological studies reported that BV and vaginal microbiome dysbiosis were corelated with STI risk (7, 8, 115, 116). However, there are no studies that demonstrate an underlying mechanism to clearly explain the relationship between BV and STI risk. In the absence of knowledge regarding an underlying mechanism, several studies have provided us with insights from which we can propose potential mechanistic explanations. As mentioned, BV-associated microbiome modulates host immune response (100–102). In our previous publication, we demonstrated that antibiotic-induced dysbiosis impairs antiviral response in a mouse HSV-2 model (117). In our report, vaginal microbiome dysbiosis markedly increased vaginal IL-33 secretion. IL-33 directly inhibited recruitment of effector CD4 and CD8 T cell and local interferon (IFN)-γ production during HSV infection. IL-33 is an alarmin released from the epithelium in response to tissue injury (118). Given some BV-related bacteria cause epithelial damage, IL-33 could be a potential candidate to explain the relationship between BV and viral infection. There was also a study suggested mechanistic explanation in HIV acquisition. Gosmann et al. prospectively followed 236 HIV-uninfected African women (119) and found women with a diverse microbial community are at 4-fold higher risk of HIV infection. Moreover, they also demonstrated participants with a diverse microbial community have more CD4 T cells, the primary target cell for HIV (120), in the vagina compared to participants with *Lactobacillus* dominated community. The author confirmed these findings in a mouse model and suggested that non-optimal vaginal microbiome increased HIV risk by inducing HIV target cell recruitment.

As with other STIs, the relationship between HPV and vaginal microbiota is complex. HPV infection is the most common viral infection in the FRT (121). Although more than 90% of HPV infection are spontaneously resolved (122), HPV infection (especially high-risk HPV 16, 18) can cause cervical intraepithelial neoplasia (CIN) that consequently develops into cervical cancer (123). Emerging evidence has begun to highlight the association between vaginal microbial community and HPV status. In a large meta-analysis study, vaginal microbiota dominated by non- *Lactobacillus* species or *Lactobacillus iners* were associated with HPV infection compared to *Lactobacillus crispatus* dominated community (9). In addition to HPV prevalence, another report demonstrated that vaginal microbiota can also contributes to CIN regression. Mitra et al. longitudinally followed young women between the ages of 16-26 who have been diagnosed with CIN2 (124). They found women with a *Lactobacillus* dominant community were more likely to have regression, whereas women with *Megasphaera, Prevotella, Gardnerella*, and BVAB1 tended to have persistent CIN2. Despite this result, the relationship between CIN and the vaginal microbiome seems to have a causality issue. In contrast to the idea that the vaginal microbiome modulates CIN progression, Kyrgiou et al. suggested a hypothesis in their review that CIN actually modulates the vaginal microbiome (125). Kyrgiou and colleagues tried to confirm their hypothesis, but they published that CIN treatment by surgical excision does not affect vaginal microbiome (126). On the contrary, another group reported that surgical treatment of CIN modulates the vaginal microbiome, resulting in an increased CST-I and concomitant decrease of CST-IV (127). To address this controversy, meta-analysis of the effect of CIN treatment on the vagina microbiome is currently in progress (128). There are also studies that reveal an association between other gynecologic cancers and the vaginal microbiome regardless of oncovirus. A recent study demonstrated that women with ovarian cancer were less likely to have a *Lactobacillus* dominated vaginal microbiome compare to those without cancer (10). This study also revealed that low abundance of *Lactobacillus* is closely related to BRCA mutation, which is a significant risk factor for ovarian cancer development (129). This result may indicate that host genetic factors regulate the composition of the vaginal microbiome. It also implies that the contribution of ovarian cancer or the BRCA mutation on the vaginal microbiome is still unclear.

### Vaginal Microbiome and Pregnancy

Traditionally, intrauterine inflammation has been considered a risk factor for poor obstetric outcomes. Because pathogens may ascended from the vagina and cause intrauterine inflammation (130), numerous studies have attempted to determine whether vaginal microbiome dysbiosis contributes to obstetric diseases. These studies revealed that vaginal microbiome dysbiosis affected the entire course of pregnancy from conception to delivery. Among the broad spectrum of obstetric diseases, preterm birth (PTB) is one of the most well-known diseases associated with vaginal microbiome dysbiosis. In one study using 16r RNA sequencing-based analysis, DiGiulio et al. reported that CST-IV inversely related with gestational age at delivery (131). The Multi-Omics Microbiome Study: Pregnancy Initiative (MOMS-PI), one of the HMP studies, supported this result (11). The MOMS-PI results showed women who delivered at term were more likely to have a *Lactobacillus crispatus* dominant community. Whereas women with PTB had higher prevalence of specific taxa including BVAB1*, Prevotella*, and *Sneathia amnii*. In addition, PTB samples had elevated levels of proinflammatory cytokines, including eotaxin, IL-1β, IL-6, and MIP-1β. Also, early pregnancy loss was associated with the vaginal microbial community. One prospective case-controlled study reported that first trimester miscarriage was associated with reduced prevalence of *Lactobacillus* species and higher alpha diversity (132). There were also studies that showed an association between the vaginal microbiome and fertility. In one study profiling the vaginal microbiome of idiopathic infertile women, the vaginal microbiome of infertile women was more similar to that of BV women compared to a healthy control (12). Furthermore, the vaginal microbiome of idiopathic infertile women had a low prevalence of *Lactobacillus* species. In line with this result, Koedooder et al. suggested the composition of vaginal microbiome could be used as a predictor of successful *in vitro* fertilization (IVF) (133). In this report, women with a low abundance of *Lactobacillus* were less likely to have a successful implantation.

Because poor obstetric outcomes are thought to be related to intrauterine inflammation, the presence and role of the endometrial microbiome have also been considered. Because bacteria did not grow in endometrial tissue, as revealed in early cultivation method-based studies (134), it was been believed the uterine cavity was sterile. However, emerging molecular-based studies highlighted that the upper FRT harbor microbiome (135). Several studies reported that endometrial microbiome is also dominated by *Lactobacillus* and that reduced prevalence of *Lactobacillus* species in the endometrium is associated with poor pregnancy outcomes (136, 137). However, because most of these results were derived from transcervical sampling, contamination from the cervix or vagina cannot be excluded. A recent study analyzed the microbiome of endometrial tissue sampled during abdominal hysterectomy (138). In this report, the endometrial microbiome was dominated by *Acinetobacter, Pseudomo*nas, and *Cloacibacterium*, whereas *Lactobacillus* species were rarely observed in the endometrium. Though the existence of an endometrial microbiome is agreed upon, the endometrial microbiome composition remains controversial. Moreover, it is unclear whether the vagina microbiome shapes the endometrial microbiome and whether the endometrial microbiome determines obstetric outcomes. Further studies are needed to elucidate the relationship between two milieus.

## Modulation of the Vaginal Microbiome by Antibiotics and Probiotics

BV symptoms, which include vaginal discharge and odor, cause discomfort. Importantly, BV is a risk factor for various diseases. Though antibiotics have been the gold standard treatment for BV, BV has a high recurrence rate following antibiotic treatment (114). As an alternative to or combination treatment with antibiotics, several methods have been proposed to restore the vaginal microbiome into a *Lactobacillus* dominated community. Oral probiotics are considered a promising method because of crosstalk between the gut and vaginal microbiomes. Many *Lactobacillus* species including *Lactobacillus rhamnosus* GR-1 and *Lactobacillus fermentum* RC-14 were tested. These oral probiotics successfully modulated the vaginal microbiome effects in reproductive and postmenopausal women (139, 140). However, a recent study indicated that there was no effect in pregnant women (141). It is necessary to evaluate the long-term efficacy of probiotics in addition to the vaginal microbiome modulation effect in pregnant women. Vaginal microbiome transplantation is another approach that has been considered. A recent publication demonstrated a successful clinical transplantation of a healthy vaginal microbiota into intractable BV patients (142). Although no placebo control was included in this study, 4 out of 5 patients had long-term remission of up to 21 months. More recently, molecular targets that may modulate the vaginal microbiome have been proposed. Bloom et al. suggested that cysteine dependence of *Lactobacillus iners* could be a potential target for vaginal microbiome modulation (143). In this study, the authors found *Lactobacillus iners* requires cysteine to grow *in vitro* and that a cysteine uptake inhibitor selectively inhibits growth of *Lactobacillus iners* rather than other *Lactobacillus* species. Consequently, the combined supplementation of metronidazole and the cysteine uptake inhibitor in BV-like community cultured *in vitro* promoted a growth of *Lactobacillus crispatus*, but competitively suppressed *Lactobacillus iners*. Critically, it is necessary to evaluate the *in vivo* efficacy of the cysteine uptake inhibitor.

## Conclusion

The host and microbiome exist in a mutual relationship. The host and microbiome constantly interact each other, especially in the complex and dynamic microenvironment of vagina. The introduction of molecular-based sequencing methods revealed that *Lactobacillus* dominated community represent reproductive health and that BV is related to various aspects of several diseases. However, most of our knowledge to date is based on cross-sectional or observational studies. Due to the lack of direct experimental evidence, there are frequently causality issues in vagina microbiome research. Paradoxically, this hurdle is derived from *Lactobacillus* dominancy of human vaginal microbiome. Due to this unique property, we cannot extend animal model results to humans. Recent advances using *in vitro* co-culture systems are expected to be a potential candidate for overcoming this hurdle. The development of suitable animal models or methodologic advances could resolve current debates discussed in this review. A better understanding of the relationship between host and microbiome could provide novel targets for diagnosis and treatment of microbiome-related diseases.

## Author Contributions

MK and HL wrote the manuscript. All authors contributed to the article and approved the submitted version.

## Funding

This study was supported by the National Research Foundation of Korea (NRF-2019R1A2C2087490 and NRF-2021M3A9H3015688) funded by the Ministry of Science and ICT of Korea.

## Conflict of Interest

The authors declare that the research was conducted in the absence of any commercial or financial relationships that could be construed as a potential conflict of interest.

## Publisher’s Note

All claims expressed in this article are solely those of the authors and do not necessarily represent those of their affiliated organizations, or those of the publisher, the editors and the reviewers. Any product that may be evaluated in this article, or claim that may be made by its manufacturer, is not guaranteed or endorsed by the publisher.
